# Conditional crude probabilities of death for English cancer patients

**DOI:** 10.1038/s41416-019-0597-0

**Published:** 2019-10-11

**Authors:** Kwok F. Wong, Paul C. Lambert, Sarwar I. Mozumder, John Broggio, Mark J. Rutherford

**Affiliations:** 1 Public Health England, National Cancer Registration and Analysis Service, Birmingham, UK; 2 Biostatistics Research Group, Department of Health Sciences, University of Leicester, Leicester, UK; 3 Department of Medical Epidemiology and Biostatistics, Karolinska Instituet, Stockholm, Sweden

**Keywords:** Cancer, Scientific community

## Abstract

**Background:**

Cancer survival statistics are typically reported by using measures discounting the impact of other-cause mortality, such as net survival. This is a hypothetical measure and is interpreted as excluding the possibility of cancer patients dying from other causes. Crude probability of death partitions the all-cause probability of death into deaths from cancer and other causes.

**Methods:**

The National Cancer Registration and Analysis Service is the single cancer registry for England. In 2006–2015, 1,590,477 malignant tumours were diagnosed for breast, colorectal, lung, melanoma and prostate cancer in adults. We used a relative survival framework, with a period approach, providing estimates for up to 10-year survival. Mortality was partitioned into deaths due to cancer or other causes. Unconditional and conditional (on surviving 1-years and 5-years) crude probability of death were estimated for the five cancers.

**Results:**

Elderly patients who survived for a longer period before dying were more likely to die from other causes of death (except for lung cancer). For younger patients, deaths were almost entirely due to the cancer.

**Conclusion:**

There are different measures of survival, each with their own strengths and limitations. Careful choices of survival measures are needed for specific scenarios to maximise the understanding of the data.

## Background

Cancer survival statistics are typically reported by using measures that discount the impact of other-cause mortality, such as net survival.^1^ These are generally reported at specific time points post diagnosis in order to assess the accumulated impact of the excess mortality associated with the cancer diagnosis. The excess mortality rate refers to the mortality contribution attributable to cancer above and beyond the mortality rate that would be expected due to other causes given a patient’s characteristics. Generally, survival metrics are reported as a population average and are often age-standardised to an external reference population^2^ for comparability across both time and countries/regions.^3^ However, cancer patients are subject to competing causes of death. That is, not all patients who are diagnosed with cancer will die from their disease. This is particularly true for elderly patients and further depends on the prognosis of the specific cancer site, which is of particular importance as non-cancer mortality can sometimes exceed mortality due to cancer.

Net survival is defined as the survival that the cohort experiences due to cancer (excess) mortality alone, in the absence of all other causes of mortality.^4^ This provides a measure of cancer survival that is particularly useful for comparisons across population groups, countries and time where other-cause mortality can differ. Net survival can help policy makers in driving change for improvement in cancer healthcare. However, net survival is a hypothetical measure as cancer patients are always at risk of dying from other causes.

In contrast to the net probability of death measure (i.e., 1-net survival), the crude probability of death (which is also known as the cumulative incidence function) provides the real-world probability of death. The all-cause probability of death is partitioned into the probability of death due to cancer and death due to other causes, which reflects the true mortality of patients in the real world.^5–10^

Conditional crude probability estimates give an updated prognosis for patients who have survived for a given time period following diagnosis.^11–13^ These measures are much more informative for patients who have already survived beyond the initial period following diagnosis and would like an updated estimate of prognosis. For these patients, in general, other-cause mortality will now play a stronger role as the cancer (excess) mortality rate diminishes over follow-up time.

The objective of this paper is to provide unconditional and conditional crude probability of death estimates for five major cancer sites in England, with a focus on showing the long-term impact of cancer on mortality for patients that have already survived for 1 or 5 years post diagnosis. Crude probabilities of death due to cancer and other causes will be presented by sex and age group, and as an example, compared with net survival estimates produced via the period approach with the same methods used to previously publish the results for the English population.^14–16^

## Methods

### Study population

The National Cancer Registration and Analysis Service (NCRAS) is run by Public Health England and is a cancer registry covering the whole of England. Tumours were classified into site-specific analyses by using the International Classification of Diseases Revision 10 (ICD10).^17^ Cancer sites included in the study were breast (C50), colorectal (C18–C20), lung (C33–C34), melanoma (C43) and prostate (C61). To produce more up-to-date estimates, a period-analysis approach was used on 839,671 patients diagnosed between 2011 and 2015 and 483,420 patients who were diagnosed between 2006 and 2010, but still alive at the start of 2011.

All patients were resident in England at the time of diagnosis and were restricted to be aged between 15 and 99 years. Patients were ineligible to enter the study if they had important missing information such as gender and vital status (Table 1). Patients were excluded if they had a missing or unknown vital status or death certificate only by registration. A full list of the exclusion criteria has previously been published.^18^ Vital status was ascertained from the Office of National Statistics (ONS) mortality data with follow-up information until the 1st January 2017.

**Table 1:** Characteristics of the cohort of adult patients, diagnosed between 15 and 99 years of age, during the period 2006–2015

	Breast cancer (C50), *N* (%)	Colorectal cancer (C18–C20), *N* (%)	Lung cancer (C33–C34), *N* (%)	Melanoma (C43), *N* (%)		Prostate cancer (C61), *N* (%)
*Overall patients*	422,306 (100%)	336,291 (100%)	354,881 (100%)	110,694 (100%)	*Overall patients*	366,305 (100%)
2006	38,742 (9.2%)	30,830 (9.2%)	32,747 (9.2%)	8938 (8.1%)	2006	31,903 (8.7%)
2007	38,764 (9.2%)	31,508 (9.4%)	32,927 (9.3%)	9086 (8.2%)	2007	32,072 (8.8%)
2008	40,722 (9.6%)	32,937 (9.8%)	34,221 (9.6%)	10,058 (9.1%)	2008	32,972 (9.0%)
2009	40,662 (9.6%)	33,471 (10.0%)	34,114 (9.6%)	10,193 (9.2%)	2009	36,249 (9.9%)
2010	41,718 (9.9%)	33,885 (10.1%)	34,842 (9.8%)	10,882 (9.8%)	2010	36,356 (9.9%)
2011	41,938 (9.9%)	34,874 (10.4%)	35,857 (10.1%)	11,201 (10.1%)	2011	36,771 (10.0%)
2012	43,039 (10.2%)	35,310 (10.5%)	37,252 (10.5%)	11,510 (10.4%)	2012	38,083 (10.4%)
2013	44,771 (10.6%)	34,346 (10.2%)	37,412 (10.5%)	12,409 (11.2%)	2013	41,355 (11.3%)
2014	46,166 (10.9%)	34,334 (10.2%)	37,868 (10.7%)	13,061 (11.8%)	2014	40,234 (11.0%)
2015	45,784 (10.8%)	34,796 (10.4%)	37,641 (10.6%)	13,356 (12.1%)	2015	40,310 (11.0%)
*Gender*					*Gender*	
Male	–	186,336 (55.4%)	195,712 (55.2%)	54,040 (48.8%)	Male	366,296 (100%)
Female	422,306 (100%)	149,955 (44.6%)	159,166 (44.8%)	56,653 (51.2%)	Female	–
*Age group*					*Age group*	
15–44	42,277 (10.0%)	10,451 (3.1%)	3938 (1.1%)	20,204 (18.3%)	15–54	14,048 (3.8%)
45–54	87,962 (20.8%)	21,859 (6.5%)	17,826 (5.0%)	16,911 (15.3%)	55–64	75,468 (20.6%)
55–64	97,004 (23.0%)	59,222 (17.6%)	62,494 (17.6%)	21,491 (19.4%)	65–74	141,431 (38.6%)
65–74	91,218 (21.6%)	96,267 (28.6%)	113,249 (31.9%)	24,264 (21.9%)	75–84	103,140 (28.2%)
75–99	103,508 (24.5%)	148,172 (44.1%)	157,222 (44.3%)	27,706 (25.0%)	85–99	32,101 (8.8%)
*Exclusions*					*Exclusions*	
Unknown gender	0 (0.0%)	0 (0.0%)	3 (0%)	0 (0.0%)	Unknown gender	0 (0.0%)
Invalid dates	3 (0.0%)	6 (0.0%)	9 (0%)	0 (0.0%)	Invalid dates	0 (0.0%)
Missing age	337 (0.1%)	320 (0.1%)	152 (0%)	118 (0.1%)	Missing age	117 (0.0%)
Invalid sex-site combination	0 (0.0%)	0 (0.0%)	0 (0%)	0 (0.0%)	Invalid sex-site combination	9 (0.0%)
DCO only	942 (0.2%)	1600 (0.5%)	2810 (0.8%)	80 (0.1%)	DCO only	1740 (0.5%)
Unknown vital status	334 (0.1%)	191 (0.1%)	107 (0.0%)	71 (0.1%)	Unknown vital status	279 (0.1%)

### Statistical analyses

Patients were included in a relative survival analysis incorporating the expected population mortality rates with a period approach to analyse the most recent available data and provide predictions for up to 10-year survival. The at-risk period began for patients who entered the period window of interest between 2011 and 2015; this includes patients diagnosed prior to 2011. Individual patient-level data were used; therefore, exact survival times were available. Estimates are presented in five age groups according to the International Cancer Survival Standard (ICSS)^19^; 15–44, 45–54, 55–64, 65–74 and 75–99 for breast, colorectal and lung and 15–54, 55–64, 65–74, 75–84 and 85–99 for prostate cancer. Only the first recorded cancer at each anatomical site was included.

Crude probabilities of death for cancer and other causes were plotted against time since diagnosis, which were calculated by the approach detailed by Cronin and Feuer.^9^ Briefly, in each time interval, the probability of death due to other causes (calculated from population lifetables stratified by patient characteristics such as age, sex and calendar year), and the probability of death due to cancer (calculated by using an interval-specific estimate of relative survival), are applied to the probability of being alive at the beginning of the interval. A correction is applied in order to account for events that could occur from both competing causes for a subsample of the patients in each interval. Conditional crude probabilities were also calculated—estimates were conditioned on having survived 1 and 5 years from diagnosis. Time since diagnosis was split into intervals for calculations. There were monthly intervals for the first 6 months following diagnosis, 3-month intervals from 6 months to 2 years, 6-month intervals to 5 years and then yearly intervals to 10 years from diagnosis.

Expected mortality was obtained directly from population mortality lifetables stratified by age, sex, deprivation and calendar year. The lifetables^20^ were generated by using population-level data held at ONS.

The results are presented in three sections: (i) the unconditional period analysis giving the cancer and other-cause crude probability of death up to 10 years after the initial cancer diagnosis; (ii) the period analysis conditional on surviving 1 year up to 10 years after the initial cancer diagnosis; (iii) the period analysis conditional on surviving 5 years up to 10 years after the initial cancer diagnosis.

All analyses were carried out with Stata software (version 15; Stata, College Station, TX; Computing Resource Center, Santa Monica, CA) by using the user-written command strs.^21^

## Results

A total of 1,590,477 patients were diagnosed with malignant cancer between 2006 and 2015 with 1,323,091 contributing to the analysis: 393,823 breast, 270,571 colorectal, 220,171 lung, 104,533 melanoma and 333,993 prostate cancers (see Table 1).

Melanoma patients had approximately equal distributions of male and female, and 55% of patients diagnosed with colorectal and lung cancer were male (see Table 1). Most of the patients were diagnosed in the oldest age group for all cancers considered except for prostate cancer, where the predominant age group was 65–74 years.

### Female breast cancer

**Fig. 1:**
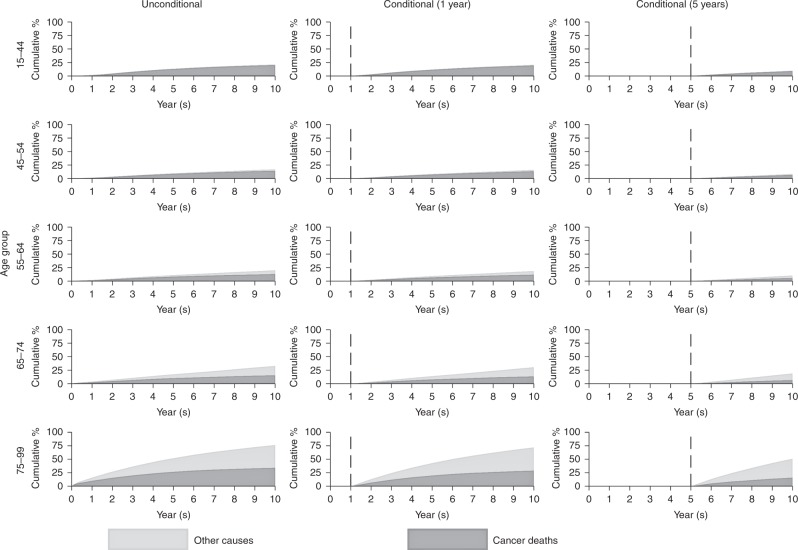
Cumulative percentage of mortality due to cancer deaths and other causes for female breast cancer

The youngest patients (aged 15–44) experience a very low probability of death due to other causes by 10 years following cancer diagnosis. For patients diagnosed at an older age, a higher proportion of deaths will be attributable to other causes. Over half of deaths in those aged between 75 and 99 are due to other causes (Fig. 1). Patients (aged 75–99 years) surviving to 1 year post diagnosis are more likely to die from other causes than cancer at 10 years. This effect is emphasised for patients who survive at least 5 years; in the oldest age group (75–99), 35.1% are predicted to die due to other causes and 15.8% due to cancer by 10 years following cancer diagnosis with the remaining 49.1% predicted to be alive.

### Colorectal cancer

**Fig. 2:**
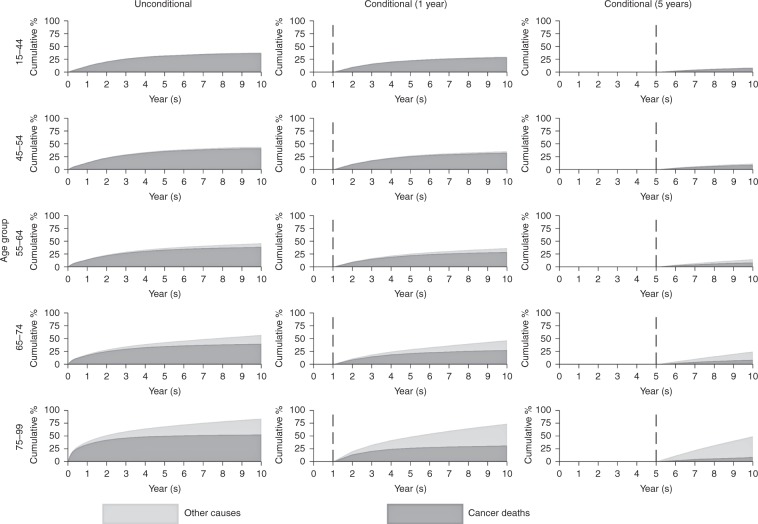
Cumulative percentage of mortality due to cancer deaths and other causes for colorectal cancer

Colorectal cancer patients experience a higher overall probability of death than breast cancer patients (Fig. 2). For patients aged 75–99 at diagnosis, the 10-year probabilities of death are 31.1% due to other causes and 52.7% due to cancer. For patients still alive 1 year after diagnosis, the proportion of patients dying from cancer drops to 31.4% at 10 years; for patients surviving to 5 years from diagnosis, the probability of dying from cancer drops to 8.9% at 10 years (whereas the proportion of patients dying from other causes is predicted to be 40.4%).

### Lung cancer

**Fig. 3:**
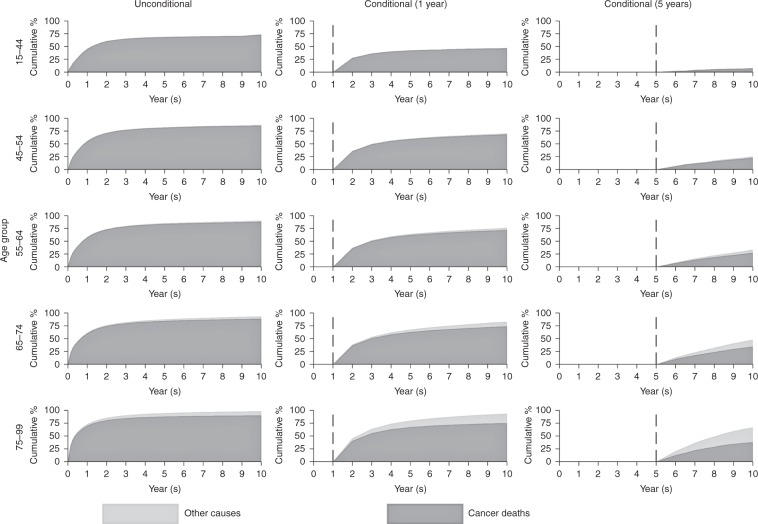
Cumulative percentage of mortality due to cancer deaths and other causes for lung cancer

Lung cancer patients experience very high mortality (Fig. 3). For patients aged 15–44 at diagnosis, 73.2% died due to cancer and 0.5% due to other causes by 10 years. In contrast, for patients aged 75–99, 90.1% died due to cancer and 8.2% due to other causes. For patients (aged 75–99 years) still alive 1 year after diagnosis, the probability of death due to cancer at 10 years dropped to 74.9%. For patients surviving to 5 years the probability of death due to cancer dropped to 37.8%.

### Melanoma

**Fig. 4:**
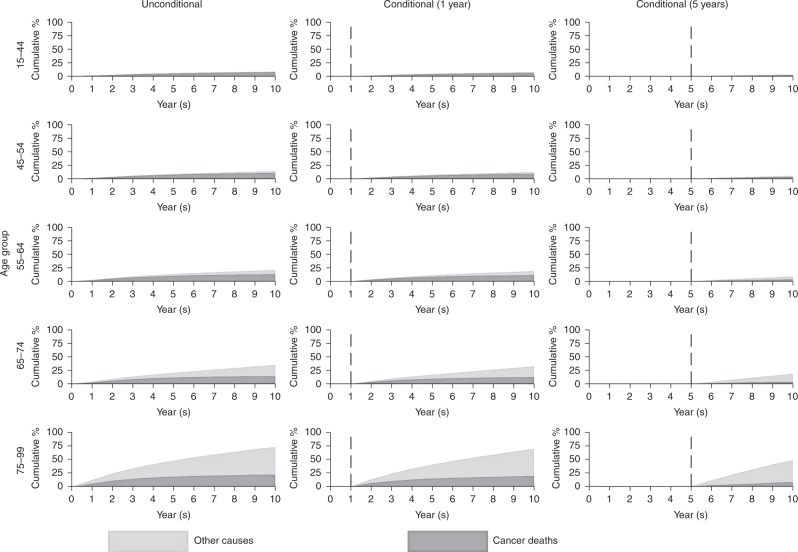
Cumulative percentage of mortality due to cancer deaths and other causes for melanoma

For patients aged 65–74 (14.1% died due to cancer and 21.0% due to other causes), the cancer mortality was lower than for patients aged 75–99 (21.8% died due to cancer and 51.0% due to other causes) at 10 years following diagnosis (Fig. 4). The predictions at 10 years post diagnosis, for patients who survived for at least 1 year (19.3% died due to cancer and 50.0% due to other causes), were similar to the unconditional estimates due to the lower impact of mortality from melanoma in the short term. In the situation where patients (aged 75–99 years) have survived for at least 5 years, the proportion of patients who die due to cancer by 10 years is further decreased (7.9%), and most of the mortality is attributed to other causes (40.6%).

### Male prostate cancer

**Fig. 5:**
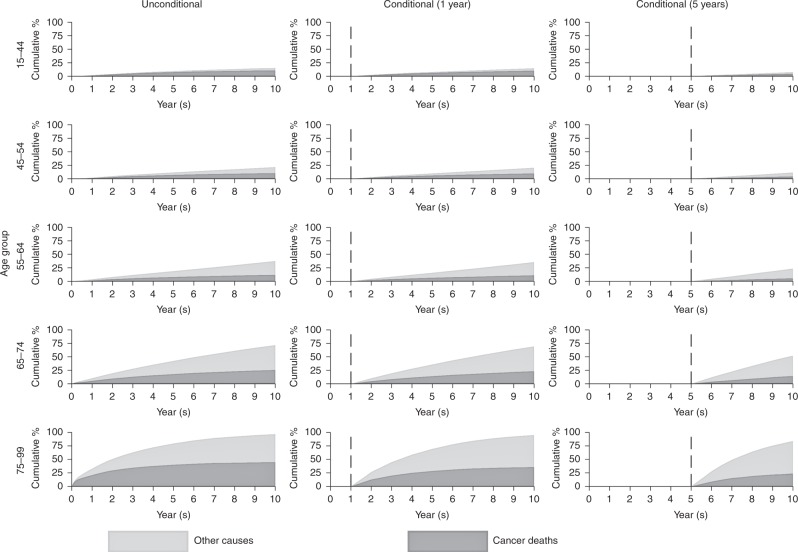
Cumulative percentage of mortality due to cancer deaths and other causes for male prostate cancer

For patients aged 85–99, 44.9% died due to cancer and 51.9% due to other causes by 10 years from cancer diagnosis (Fig. 5). The probability of death due to cancer is lower for patients who survive for at least 1 year. For patients surviving for at least 5 years, 23.7% died due to cancer and 60.3% due to other causes by 10 years from cancer diagnosis. With the exception of the youngest age group, individuals who survived for at least 1 year were more likely to die from other causes than cancer at 10 years from diagnosis.

## Discussion

Crude probabilities of death are a useful metric for reflecting the survival experience of cancer patients in the context of competing causes of death. Furthermore, conditional estimates are useful for providing updated information on the impact of cancer on mortality for those patients who have survived for a given time beyond diagnosis. It is useful to present statistics in different ways that may be better suited and understood by different audiences. Net measures, although useful for comparisons when we want to remove differences in other-cause mortality rates between comparison populations, are difficult for clinicians and patients to understand what actually happens to patients in the real world. Therefore, this study aimed to partition the all-cause probability of death of cancer patients into deaths due to cancer and other causes by using crude probabilities. This was driven by the need to more accurately reflect the true probability of death of patients as many published documents solely present net survival.^22^

It is particularly important to assess the contribution of deaths due to other causes in cancer patients when considering longer-term survival. The mortality rate due to cancer decreases over follow-up time, and the rate of mortality due to other causes will increase. The probability of death due to other causes varies according to cancer site because different cancers have different prognosis. Mortality from cancers such as melanoma (Fig. 4) will have a higher probability of death due to other causes at older ages due to good prognosis of melanoma. Whereas patients with cancers such as lung cancer (Fig. 3) have a much lower probability of death due to other causes due to the relatively poor prognosis of lung cancer patients.

‘One minus net survival’ can be contrasted with the cancer component of the crude probability of death, where one is a metric of the absence of competing causes of death and the other in the presence. Young patients have a low other-cause mortality rate, and therefore the crude and net measures are very similar (see Supplementary Table A1). There is a greater difference between the net and crude cancer measures for older age groups as the competing mortality rate due to the other causes is much greater.

Conditional measures offer further insights demonstrating that those who have survived 1 or 5 years after diagnosis now have a smaller component of their all-cause probability of death that is due to their cancer (see Supplementary Tables A2–A6). This is because the excess mortality rates decrease over follow-up time. Conditional metrics can be a further useful reporting tool when explaining the impact of cancer on mortality. These measures can be presented as natural frequency descriptions or people charts to facilitate interpretation for clinicians and patients.^23^

It is clear that using crude probabilities of death helps the understanding of cancer survival particularly in the long term. Therefore, it would be beneficial to include as part of reports and National statistics, especially for population-level statistics, with the caveat that these metrics are less comparable over time, given the changing nature of the chance of dying from causes other than cancer due to an aging cancer population.

### Limitations

We conducted and reported the results in five age groups and by 5-year aggregated periods so that the visuals match the presentation of the National Statistics for cancer survival produced yearly by the Public Health England and Office for National Statistics partnership. In practice, this could be computed for a single year of age as well as single non-aggregated years, providing more accurate estimates for specific ages or years, but would require the use of statistical modelling.^13^ Flexible parametric survival modelling was not performed here but would offer an approach to obtaining more individualised predictions. Another limitation is the inability to provide stratification by stage of the disease and comorbidity, since the prognosis of patients diagnosed with different stages varies substantially, and comorbidity will impact both causes of death. Staging data in England are not sufficiently complete historically to provide long-term stage-specific survival estimates. Stage information has improved notably since 2012, and providing longer-term stage-specific survival estimates in England will soon be possible.^24^ Mortality due to the other causes can also vary by comorbidity for individuals with the same age,^25^ and therefore stratifying lifetables by comorbidity would also be informative.

## Conclusion

Net survival is a very useful measure for estimating the effect of cancer on patient survival and is particularly suited to ensure fair comparisons of the impact of cancer across groups with differing other-cause mortality. However, crude probabilities are a more useful measure for reflecting the true probability of death as it presents mortality partitioned into deaths due to cancer and deaths due to other causes, and appropriately reports the impact of competing mortality. Careful consideration needs to be given as to which survival measure should be presented in specific scenarios to maximise the understanding of the data. Estimates of crude probabilities (including conditional) should be considered in settings where patients and clinicians are the intended audience for the statistics.

## Supplementary information

Supplementary Tables

## Data Availability

The data are available and can be provided by the Office for Data Release (ODR). The authors are employed by Public Health England operated under Section 251 of the National Health Service Act 2006 to access and analyse the data. Authors employed outside of Public Health England did not have direct access to the data but collaborated on the methodology, results and paper.
