# Supplementary material for: Conditional crude probabilities of death for English cancer patients
Source: Br J Cancer. 2019 Oct 11;121(10):883–9. doi: 10.1038/s41416-019-0597-0 (PMC6889443; doi:10.1038/s41416-019-0597-0)
Supplement: Supplementary file 1 — Supplementary Tables [file 41416_2019_597_MOESM1_ESM.pdf]

	Estimated % of deaths in the cohort, following 5 years from diagnosis of cancer			
	1 – Net Survival*	Cumulative Mortality % due to Cancer deaths (Unconditional)	Cumulative Mortality % due to Other deaths (Unconditional)	Cumulative Mortality % due to any death (Unconditional)
Breast				
15-44	13.0%	13.0%	0.5%	13.5%
45-54	8.7%	8.6%	1.1%	9.7%
55-64	8.4%	8.3%	2.9%	11.2%
65-74	10.6%	10.3%	7.1%	17.4%
75-99	31.6%	26.5%	25.3%	51.8%
Colorectal				
15-44	32.0%	32.0%	0.4%	32.4%
45-54	36.1%	36.0%	1.3%	37.3%
55-64	33.8%	33.4%	3.2%	36.6%
65-74	36.3%	35.3%	7.7%	43.0%
75-99	54.2%	50.3%	18.4%	68.7%
Lung				
15-44	68.4%	68.4%	0.3%	68.7%
45-54	82.0%	81.8%	0.6%	82.4%
55-64	84.3%	83.8%	1.3%	85.1%
65-74	85.9%	84.6%	3.0%	87.6%
75-99	92.2%	88.0%	6.7%	94.7%
Melanoma				
15-44	5.3%	5.3%	0.4%	5.7%
45-54	7.7%	7.6%	1.3%	8.9%
55-64	10.2%	10.1%	3.4%	13.5%
65-74	12.1%	11.6%	8.7%	20.3%
75-99	21.6%	18.0%	29.5%	47.5%
Prostate				
15-54	7.6%	7.5%	1.9%	9.4%
55-64	7.2%	7.1%	4.6%	11.7%
65-74	8.2%	7.8%	10.9%	18.7%
75-84	20.7%	17.9%	24.5%	42.4%
85-99	51.3%	39.9%	39.5%	79.4%

*Net survival estimates were obtained from the same analysis as those published on ONS[2], but recreated using period approach.

Table S1: Comparison of published net survival estimates to cumulative probabilities of death due to cancer, during the period 2011-2015.

	Cumulative Mortality % at **X** years following a diagnosis of cancer					
	**1 Year(s)**		**5 Year(s)**		**10 Year(s)**	
Age Group	*Cancer Deaths*	*Other Causes*	*Cancer Deaths*	*Other Causes*	*Cancer Deaths*	*Other Causes*
Breast						
Unconditional						
15-44	1.5% (1.4 – 1.7)	0.1% (0.1 – 0.1)	13.0% (12.5 – 13.5)	0.5% (0.5 – 0.5)	20.2% (19.5 – 21.0)	1.1% (1.1 – 1.1)
45-54	1.3% (1.2 – 1.4)	0.2% (0.2 – 0.2)	8.6% (8.3 – 8.9)	1.1% (1.1 – 1.1)	14.2% (13.6 – 14.8)	2.7% (2.7 – 2.7)
55-64	1.6% (1.5 – 1.7)	0.5% (0.5 – 0.5)	8.3% (8.0 – 8.6)	2.9% (2.9 – 2.9)	13.0% (12.4 – 13.6)	7.1% (7.1 – 7.1)
65-74	2.7% (2.5 – 2.9)	1.2% (1.2 – 1.2)	10.3% (9.9 – 10.6)	7.1% (7.1 – 7.2)	15.5% (14.7 – 16.4)	17.2% (17.1 – 17.2)
75-99	9.9% (9.6 – 10.3)	6.0% (5.9 – 6.0)	26.5% (26.0 – 27.1)	25.3% (25.2 – 25.4)	34.0% (33.1 – 35.0)	42.3% (42.0 – 42.6)
Breast						
Conditional 1 Year(s)						
15-44			11.6% (11.2 – 12.1)	0.4% (0.4 – 0.4)	19.7% (19.1 – 20.3)	1.0% (1.0 – 1.0)
45-54			7.4% (7.2 – 7.7)	0.9% (0.9 – 0.9)	13.5% (13.1 – 13.9)	2.6% (2.6 – 2.6)
55-64			6.8% (6.5 – 7.1)	2.5% (2.5 – 2.5)	11.9% (11.5 – 12.3)	6.7% (6.7 – 6.7)
65-74			7.8% (7.5 – 8.2)	6.1% (6.1 – 6.2)	13.6% (13.0 – 14.1)	16.8% (16.7 – 16.8)
75-99			19.8% (19.2 – 20.4)	23.0% (22.9 – 23.1)	28.8% (28.1 – 29.5)	43.1% (42.8 – 43.4)
Breast						
Conditional 5 Year(s)						
15-44					9.2% (8.7 – 9.6)	0.7% (0.7 – 0.7)
45-54					6.6% (6.3 – 6.9)	1.8% (1.8 – 1.8)
55-64					5.6% (5.3 – 5.9)	4.7% (4.7 – 4.7)
65-74					6.7% (6.2 – 7.2)	12.4% (12.3 – 12.7)
75-99					15.8% (16.7 – 35.1)	35.1% (34.9 – 35.4)

Table S2: Cumulative probability partitioned by deaths due to cancer and other causes, during the period 2011 to 2015, for female breast cancer patients.

	Cumulative Mortality % at **X** years following a diagnosis of cancer					
	**1 Year(s)**		**5 Year(s)**		**10 Year(s)**	
Age Group	*Cancer Deaths*	*Other Causes*	*Cancer Deaths*	*Other Causes*	*Cancer Deaths*	*Other Causes*
Colorectal						
Unconditional						
15-44	12.0% (11.1 – 12.8)	0.1% (0.1 – 0.1)	32.0% (30.7 – 33.3)	0.4% (0.4 – 0.4)	36.9% (35.2 – 38.5)	1.0% (1.0 – 1.0)
45-54	13.5% (12.9 – 14.2)	0.3% (0.3 – 0.3)	36.0% (35.0 – 36.6)	1.3% (1.2 – 1.3)	41.1% (39.9 – 42.3)	2.8% (2.7 – 2.8)
55-64	14.3% (13.9 – 14.7)	0.6% (0.6 – 0.6)	33.4% (32.9 – 34.0)	3.2% (3.2 – 3.2)	38.9% (38.0 – 39.8)	7.2% (7.2 – 7.3)
65-74	17.9% (17.5 – 18.2)	1.6% (1.6 – 1.6)	35.3% (34.8 – 35.8)	7.7% (7.6 – 7.7)	40.1% (39.3 – 40.9)	16.9% (16.7 – 17.0)
75-99	34.0% (33.6 – 34.4)	5.1% (5.1 – 5.2)	50.3% (49.8 – 50.7)	18.4% (18.3 – 18.5)	52.7% (52.0 – 53.4)	31.1% (30.9 – 31.4)
Colorectal						
Conditional 1 Year(s)						
15-44			22.8% (21.5 – 24.0)	0.4% (0.4 – 0.4)	28.9% (27.4 – 30.3)	1.0% (1.0 – 1.1)
45-54			26.0% (25.1 – 26.9)	1.2% (1.1 – 1.2)	32.6% (31.6 – 33.7)	2.9% (2.9 – 3.0)
55-64			22.5% (22.0 – 23.1)	3.0% (3.0 – 3.0)	28.8% (28.1 – 29.5)	7.7% (7.6 – 7.7)
65-74			21.6% (21.1 – 22.1)	7.6% (7.6 – 7.6)	27.7% (27.1 – 28.3)	19.0% (18.8 – 19.1)
75-99			26.8% (26.2 – 27.3)	21.8% (21.7 – 21.9)	31.4% (30.6 – 32.1)	42.6% (42.3 – 42.9)
Colorectal						
Conditional 5 Year(s)						
15-44					7.9% (6.8 – 9.1)	0.9% (0.8 – 0.9)
45-54					9.1% (8.2 – 10.0)	2.4% (2.4 – 2.4)
55-64					8.4% (7.8 – 9.0)	6.2% (6.2 – 6.3)
65-74					8.6% (8.0 – 9.3)	16.1% (16.0 -16.1)
75-99					8.9% (8.0 – 9.9)	40.4% (40.1 – 40.7)

Table S3: Cumulative mortality partitioned by deaths due to cancer and other causes, during the period 2011 to 2015, for colorectal cancer patients.

	Cumulative Mortality % at **X** years following a diagnosis of cancer					
	**1 Year(s)**		**5 Year(s)**		**10 Year(s)**	
Age Group	*Cancer Deaths*	*Other Causes*	*Cancer Deaths*	*Other Causes*	*Cancer Deaths*	*Other Causes*
Lung						
Unconditional						
15-44	45.4% (43.1 – 47.6)	0.1% (0.1 – 0.1)	68.4% (66.2 – 70.5)	0.3% (0.3 – 0.3)	73.2% (69.4 – 76.7)	0.5% (0.5 – 0.5)
45-54	55.4% (54.4 – 56.5)	0.2% (0.2 – 0.2)	81.8% (81.0 – 82.7)	0.6% (0.6 – 0.6)	85.9% (84.8 – 87.0)	1.0% (1.0 – 1.0)
55-64	57.9% (57.3 – 58.5)	0.5% (0.5 – 0.5)	83.8% (83.4 – 84.2)	1.3% (1.3 – 1.3)	88.1% (87.5 – 88.7)	2.2% (2.2 – 2.3)
65-74	60.4% (60.0 – 60.8)	1.1% (1.1 – 1.1)	84.6% (84.2 – 84.9)	3.0% (2.9 – 3.0)	88.7% (88.2 – 89.2)	4.6% (4.6 – 4.7)
75-99	69.9% (69.6 – 70.3)	3.3% (3.3 – 3.3)	88.0% (87.7 – 88.2)	6.7% (6.6 – 6.8)	90.1% (89.8 – 90.4)	8.2% (8.1 – 8.3)
Lung						
Conditional 1 Year(s)						
15-44			42.2% (39.1 – 45.3)	0.3% (0.3 – 0.3)	46.5% (43.2 – 49.7)	0.8% (0.8 – 0.9)
45-54			59.5% (57.9 – 61.1)	0.9% (0.8 – 0.9)	68.6% (67.0 – 70.3)	1.8% (1.8 – 1.9)
55-64			62.3% (61.4 – 63.2)	2.0% (2.0 – 2.1)	72.2% (71.2 – 73.1)	4.1% (4.0 – 4.2)
65-74			62.8% (62.1 – 63.5)	4.8% (4.8 – 4.9)	73.9% (73.1 – 74.7)	9.2% (9.1 – 9.4)
75-99			67.4% (66.6 – 68.1)	12.6% (12.4 – 12.8)	74.9% (74.2 – 75.7)	18.4% (18.1 – 18.8)
Lung						
Conditional 5 Year(s)						
15-44					7.5% (5.1 – 10.4)	0.8% (0.8 – 0.9)
45-54					23.1% (20.5 – 25.7)	2.5% (2.4 – 2.5)
55-64					27.7% (25.9 – 29.6)	5.9% (5.8 – 6.0)
65-74					34.4% (32.5 – 36.2)	13.5% (13.3 – 13.8)
75-99					37.8% (35.0 – 40.5)	29.0% (28.3 – 29.8)

Table S4: Cumulative mortality partitioned by deaths due to cancer and other causes, during the period 2011 to 2015, for lung cancer patients.

	Cumulative Mortality % at **X** years following a diagnosis of cancer					
	**1 Year(s)**		**5 Year(s)**		**10 Year(s)**	
Age Group	*Cancer Deaths*	*Other Causes*	*Cancer Deaths*	*Other Causes*	*Cancer Deaths*	*Other Causes*
Melanoma<br>*Unconditional*						
15-44	1.3% (1.1 – 1.5)	0.1% (0.1 – 0.1)	5.3% (4.9 – 5.8)	0.4% (0.4 – 0.4)	7.6% (6.8 – 8.3)	1.1% (1.1 – 1.1)
45-54	1.5% (1.2 – 1.7)	0.2% (0.2 – 0.2)	7.6% (7.0 – 8.2)	1.3% (1.3 – 1.3)	10.7% (9.6 – 11.9)	3.2% (3.1 – 3.2)
55-64	2.0% (1.7 – 2.3)	0.6% (0.6 – 0.6)	10.1% (9.4 – 10.8)	3.4% (3.4 – 3.4)	13.3% (12.0 – 14.6)	8.2% (8.1 – 8.2)
65-74	2.5% (2.2 – 2.9)	1.6% (1.6 – 1.6)	11.6% (10.8 – 12.4)	8.7% (8.7 – 8.8)	14.1% (12.4 – 16.0)	21.0% (20.8 – 21.2)
75-99	5.2% (4.7 – 5.8)	6.9% (6.9 – 6.9)	18.0% (17.0 – 19.1)	29.5% (29.2 – 29.7)	21.8% (19.7 – 23.9)	51.0% (50.4 – 51.7)
Melanoma<br>*Conditional 1 Year(s)*						
15-44			4.1% (3.7 – 4.5)	0.4% (0.4 – 0.4)	6.5% (5.9 – 7.0)	1.0% (1.0 – 1.0)
45-54			6.3% (5.7 – 6.9)	1.1% (1.1 – 1.1)	9.3% (8.5 – 10.1)	3.0% (3.0 – 3.0)
55-64			8.3% (7.7 – 9.0)	2.9% (2.8 – 2.9)	11.5% (10.7 – 12.4)	7.7% (7.6 – 7.7)
65-74			9.4% (8.7 – 10.2)	7.5% (7.5 – 7.5)	12.5% (11.4 – 13.7)	20.0% (19.9 – 20.2)
75-99			14.6% (13.5 – 15.7)	25.7% (25.5 – 25.9)	19.3% (17.7 – 20.9)	50.0% (49.3 – 50.6)
Melanoma<br>*Conditional 5 Year(s)*						
15-44					2.5% (2.1 – 2.9)	0.7% (0.7 – 0.7)
45-54					3.3% (2.7 – 4.0)	2.1% (2.1 – 2.1)
55-64					3.6% (2.9 – 4.4)	5.4% (5.4 – 5.5)
65-74					3.7% (2.7 – 4.9)	15.1% (15.0 – 15.2)
75-99					7.9% (6.4 – 10.1)	40.6% (40.1 – 41.1)

Table S5: Cumulative mortality partitioned by deaths due to cancer and other causes, during the period 2011 to 2015, for melanoma patients.

	Cumulative Mortality % at **X** years following a diagnosis of cancer					
	**1 Year(s)**		**5 Year(s)**		**10 Year(s)**	
Age Group	*Cancer Deaths*	*Other Causes*	*Cancer Deaths*	*Other Causes*	*Cancer Deaths*	*Other Causes*
Prostate						
Unconditional						
15-54	1.4% (1.2 – 1.8)	0.3% (0.3 – 0.3)	7.5% (6.8 – 8.2)	1.9% (1.9 – 1.9)	10.5% (9.2 – 11.8)	4.8% (4.7 – 4.8)
55-64	1.2% (1.0 – 1.3)	0.8% (0.8 – 0.8)	7.1% (6.7 – 7.4)	4.6% (4.6 – 4.6)	10.3% (9.6 – 11.1)	11.2% (11.2 – 11.2)
65-74	1.7% (1.5 – 1.8)	1.9% (1.9 – 1.9)	7.8% (7.5 – 8.1)	10.9% (10.9 – 11.0)	11.9% (11.2 – 12.6)	25.6% (25.5 – 25.7)
75-84	5.4% (5.1 – 5.6)	5.0% (5.0 – 5.0)	17.9% (17.4 – 18.4)	24.5% (24.4 – 24.6)	25.5% (24.5 – 26.5)	46.1% (45.8 – 46.4)
85-99	20.9% (20.1 – 21.7)	11.8% (11.8 – 11.9)	39.9% (38.8 – 41.0)	39.5% (38.9 – 40.0)	44.9% (43.5 – 46.3)	51.9% (50.9 – 52.8)
Prostate						
Conditional 1 Year(s)						
15-54			6.2% (5.6 – 6.8)	1.6% (1.6 – 1.6)	10.4% (9.4 – 11.4)	4.5% (4.5 – 4.5)
55-64			6.0% (5.7 – 6.3)	3.9% (3.9 – 3.9)	9.8% (9.4 – 10.3)	10.6% (10.5 – 10.6)
65-74			6.4% (6.1 – 6.7)	9.4% (9.3 – 9.4)	11.0% (10.5 – 11.4)	24.5% (24.4 – 24.6)
75-84			14.0% (13.5 – 14.5)	21.8% (21.7 – 21.8)	23.2% (22.5 – 24.0)	46.0% (45.7 – 46.2)
85-99			28.3% (26.9 – 29.7)	41.1% (40.5 – 41.6)	35.6% (34.1 – 37.0)	59.5% (28.4 – 60.7)
Prostate						
Conditional 5 Year(s)						
15-54					4.6% (3.8 – 5.5)	3.1% (3.1 – 3.1)
55-64					4.2% (3.8 – 4.6)	7.4% (7.4 – 7.4)
65-74					5.5% (5.0 – 5.9)	18.0% (18.0 – 18.1)
75-84					14.4% (13.5 – 15.2)	37.7% (37.4 – 37.9)
85-99					23.7% (20.7 – 26.8)	60.3% (58.4 – 62.0)

Table S6: Cumulative mortality partitioned by deaths due to cancer and other causes, during the period 2011 to 2015, for male prostate cancer patients.
